# Risk stratification in patients with upper gastrointestinal submucosal tumors undergoing submucosal tunnel endoscopic resection

**DOI:** 10.3389/fmed.2022.1067576

**Published:** 2022-12-22

**Authors:** Yong Lv, Shaohua Li, Xiuhe Lv, Qing Liu, Yu Zheng, Yang Su, Changbin Yang, Yanglin Pan, Liping Yao, Huahong Xie

**Affiliations:** ^1^State Key Laboratory of Cancer Biology, National Clinical Research Center for Digestive Diseases and Xijing Hospital of Digestive Diseases, Fourth Military Medical University, Xi’an, China; ^2^Military Medical Innovation Center, Fourth Military Medical University, Xi’an, China

**Keywords:** submucosal tunnel, endoscopic resection (ER), submucosal tumor (SMT), risk stratification, procedure-related complications

## Abstract

**Background:**

A substantial heterogeneity exists in patients with upper gastrointestinal submucosal tumors (SMTs). This study aimed to identify predictors of long procedure time (≥60 min), occurrence of procedure-related complications, and long hospital stay (≥6 days) in patients with SMTs undergoing submucosal tunnel endoscopic resection (STER) and stratify risk based on the predictors.

**Methods:**

Sixty-six consecutive patients with upper gastrointestinal SMTs undergoing STER between January 2013 and December 2018 were retrospectively included. Binary logistic regression models were developed to identify predictors of outcomes. Receiver operating characteristic (ROC) curves were constructed to evaluate the discrimination of tumor size.

**Results:**

Complete resection and *en bloc* resection of tumor were achieved in 66 (100%) and 64 patients (97%), respectively. Twenty-seven patients (41%) had a long procedure time, 10 (15%) developed STER-related complications, and 17 (26%) had a long hospital stay. On multivariable analysis, tumor size was an independent predictor of long procedure time (OR 1.37, 95% CI 1.13–1.67; *p* = 0.001), occurrence of complications (OR 1.06, 95% CI 1.01–1.10; *p* = 0.012), and long hospital stay (OR 1.05, 95% CI 1.01–1.09; *p* = 0.035). ROC curves identified a tumor of size 25 mm as the best cutoff; those who had a tumor above this value had a 76-fold risk of long procedure time, 8.56-fold risk of occurrence of complications, and 6.35-fold risk of long hospital stay.

**Conclusion:**

Patients with a tumor size ≥25 mm had longer procedure time, higher risk of STER-related complications, and longer hospital stay; therefore, they should be classified as a high-risk group.

## Introduction

Gastrointestinal submucosal tumors (SMTs) are a class of protruding lesions with a normal mucosa-covered surface, which are commonly discovered during endoscopic examination (1). With the advancement of new endoscopic techniques, the detection rate of SMTs was greatly increased in recent years (1). Although most SMTs are benign, long-term surveillance may increase both the financial burden and mental stress for patients (2). Furthermore, some SMTs, especially those originating from the muscularis propria (MP) or with a large diameter, are potentially malignant (3). The elimination of malignancy seems difficult without resection (4).

Conventional surgery and therapeutic endoscopy are current therapies for SMTs. Surgery has previously been the primary treatment option for SMTs but proved to be more invasive and time-consuming than endoscopic resection. In recent years, various endoscopic therapies have been used for the treatment of upper gastrointestinal SMTs, which can provide both definitive histological diagnosis and a minimally invasive therapeutic approach to such tumors (5). Among them, submucosal tunnel endoscopic resection (STER) is an emerging technology that uses a submucosal tunnel as an operating space to resect targeted tumors with the advantage of maintaining the integrity of digestive tract mucosa (6–8). Previous studies have shown that STER for upper gastrointestinal SMTs originating from the MP layer was effective and safe (5, 9, 10). However, the reported procedure time, incidence of complications, and hospital time vary widely among studies (5, 9). This variability suggests that cohorts included in these studies belong to different risk subgroups. Indeed, substantial heterogeneity exists in the patients with upper gastrointestinal SMTs with regard to patient demography (age and gender) and tumor characteristics (size, location, shape, histopathology, etc.). Therefore, further risk stratification is necessary to identify patients with high-risk and to guide individualized treatment. Nevertheless, no such study has been published.

Thus, the aim of this study was to investigate the risk factors associated with the long procedure time, occurrence of STER-related complications, and long hospital stay in patients with upper gastrointestinal SMTs undergoing STER and make risk stratification based on those factors.

## Patients and methods

### Study design and participants

We retrospectively extracted the data from the electronic charts of consecutive patients with upper gastrointestinal SMTs who were treated by STER at Xijing Hospital (a tertiary university hospital in China) from January 2013 to December 2018. This retrospective study was approved by the ethics committee of Xijing Hospital, and informed consent was got from all patients or their next of kin.

The inclusion criteria for the present study were (1) diagnosis with upper gastrointestinal SMTs confirmed by computed tomography (CT) and endoscopic ultrasound (EUS) and (2) receiving STER treatment. Patients with more than one SMTs, previous endoscopic resection of submucosal tumors, previous peroral endoscopic myotomy (POEM), or incomplete baseline data were excluded.

The primary endpoint for the study was the long procedure time which was defined as the overall procedure time being 60 min or more (6). The procedure of STER includes three stages: tunnel formation (the beginning of mucosal incision to the establishment of the tunnel), tumor resection (the beginning of resection to the removal of tumors), and tunnel closing (the beginning of reparation to the closure of tunnel). The time of each stage was recorded and calculated altogether as the overall operation time (that is the period from mucosal incision to the closure of mucosal incision).

The secondary outcomes include the occurrence of STER-related complications and a long hospital stay (6 days or more). The STER-related complication was defined as any adverse events related to the procedure, including air leakage symptoms (subcutaneous emphysema, pneumomediastinum, and pneumothorax), esophageal-pleural fistula, perforation, mucosal injury, muscular injury, acute or delayed major bleeding, gastrointestinal tract leakage, or secondary peritoneal/abdominal infections (8).

### STER procedure

All procedures were performed with patients under propofol general anesthesia by two operators with rich experience in performing peroral endoscopic myotomy (POEM) and endoscopic submucosal dissection (ESD).

**FIGURE 1:**
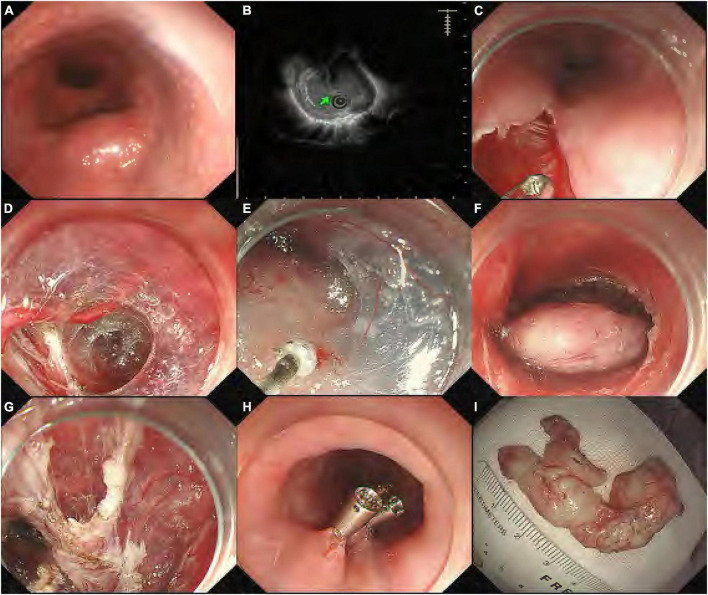
Submucosal tunneling endoscopic resection procedure for a submucosal tumor (SMT) from the muscularis propria (MP) layer. **(A)** Endoscopic view of the esophageal SMT; **(B)** endoscopic ultrasonography showing the SMT originating from the MP layer; **(C)** a longitudinal mucosal incision was made; **(D)** endoscopy showing the formation of the submucosal tunnel with pre-coagulation to visible vessels; **(E)** dissection of the tumor from surrounding submucosal tissue; **(F)** the entire exposed tumor after muscularis dissection; **(G)** endoscopy showing the esophageal MP defect without perforation; **(H)** the mucosal entry incision was closed with clips; and **(I)** the resected specimen was a 4.5-cm leiomyoma.

Before the procedure, enhanced computed tomography (CT) and endoscopic ultrasonography (EUS) were adopted in all patients who were suspected of having upper gastrointestinal SMTs, in order to evaluate the location, size, shape, and depth of the tumor and eliminate metastasis or invasion outside the digestive tract. The STER procedure has been described elsewhere, and Figure 1 shows the key steps. In brief, a submucosal fluid cushion was first made at 2 to 6 cm proximal to the SMT. Following a 2-cm mucosal incision on the mucosal surface, a longitudinal tunnel ending 1–2 cm distal to the tumor between the submucosal and muscular layers was established with a hybrid knife (ERBE) or the hook knife (Olympus). After the tumor was resected and complete hemostasis in the submucosal tunnel was confirmed, the incision was closed with several clips (HX-610-135; Olympus). Complete resection was considered when the tumor was removed completely with negative margins, while *en bloc* resection was defined as the complete removal of the tumor into one non-fragmented piece (5). After the procedure, all patients were closely monitored with a complete blood count examination the next morning. Oral intake of food was restarted 48 h after STER, and an intravenous proton pump inhibitor (PPI) and antibiotics were given for 3 days, followed by a 4-week oral PPI therapy.

### Follow-up

After discharge, patients were followed with endoscopy at 1, 3, 6, and 12 months after the procedure to observe wound healing and to detect residual lesions. The EUS was performed to check for any residual lesions in the 6th month. Subsequently, endoscopy and EUS were performed to screen local recurrent lesions, and CT was used to check distant metastasis annually. Recurrence was considered if SMTs were found within 1.0 cm around primary resected lesions more than 6 months after STER, while residual was regarded as redetection of SMTs within 1.0 cm around primary resected lesions less than 6 months after STER (8).

### Statistical analysis

Continuous variables were summarized as median (range) and compared using the non-parametric Mann–Whitney *U*-test. Categorical values were shown as numbers (percentages) and compared using the chi-squared test or Fisher’s exact test wherever appropriate. Stepwise logistic regression analysis was used to identify independent predictors for long procedure time, occurrence of complications, and long hospital stay. Comparisons between patients with and without long procedure time, occurrence of complications, and long hospital stay were first performed by using univariable logistic analysis. Multivariable analysis with backward stepwise logistic regression analysis was performed with the variables that had attained a *p*-value of <0.1 on univariable analysis. The association of each variable with the evaluated endpoint was reported as an odds ratio (OR) with a 95% confidence interval (CI). The restricted cubic splines were adopted to visualize the non-linear relationships between the tumor size and the evaluated outcomes by entering the tumor size as a continuous variable into the logistic regression models. The area under the curve (AUC) of the receiver operating characteristic (ROC) curve was used to evaluate the ability of the tumor size to predict long procedure time, occurrence of complications, and long hospital stay. The Youden index (sensitivity + specificity-1) was used to identify the optimal cutoff point of tumor size predicting these outcomes. A statistical significance was set when a two-tailed *p*-value was less than 0.05. All statistical analyses were done with R 3.6.1^1^ software packages.

## Results

### Clinicopathological characteristics

**FIGURE 2:**
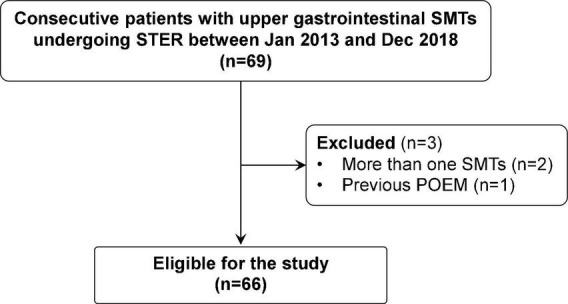
A flowchart showing the study design and patients’ disposition. POEM, peroral endoscopic myotomy; SMT, submucosal tumors; STER, submucosal tunnel endoscopic resection.

During the study period, 69 consecutive patients with upper gastrointestinal SMTs underwent STER in our center. Three patients were excluded due to more than one SMT (*n* = 2) or previous POEM (*n* = 1). Finally, 66 eligible patients were included in our study (Figure 2), including 34 women and 32 men with a median age of 53 years (range: 27–73). The clinicopathological characteristics of included patients are described in Table 1. The median size of the tumor was 18 mm (range: 6–75), which was localized in the esophagus in 34 patients (52%), esophagogastric junction in 24 patients (36%), and stomach in eight patients (12%). Most tumors originated in the muscularis propria [53 patients (80%)], followed by muscularis mucosa [11 patients (17%)], and submucosa [two patients (3%)]. The final pathological diagnosis was leiomyomas [50 patients (76%)], gastrointestinal stromal tumors [11 patients (17%)], lipomas [one patient (2%)], schwannoma [two patients (3%)], and fibroma [two patients (4%)]. No patient received anticoagulants/antiplatelet agents or had previous upper gastrointestinal surgery.

**TABLE 1:** Clinicopathological characteristics and outcomes of 66 patients with upper gastrointestinal submucosal tumors treated with submucosal tunnel endoscopic resection (STER).

Characteristics	Values (*n* = 66)
Age (years), median (range)	53 (27–73)
**Gender, *n* (%)**	
Male	32 (48%)
Female	34 (52%)
Tumor size (mm), median (range)	18 (6–75)
**Tumor location, *n* (%)**	
Upper esophagus	4 (6%)
Middle esophagus	8 (12%)
Lower esophagus	22 (33%)
Esophagogastric junction	24 (36%)
Stomach	8 (12%)
**Tumor layer, *n* (%)**	
Muscularis mucosa	11 (17%)
Submucosa	2 (3%)
Muscularis propria	53 (80%)
**Tumor shape, *n* (%)**	
Regular	51 (77%)
Irregular	15 (23%)
Tunnel length (cm), median (range)	3 (1–6)
**Histopathology, *n* (%)**	
Leiomyomas	50 (76%)
Gastrointestinal stromal tumors	11 (17%)
Lipomas	1 (2%)
Schwannoma	2 (3%)
Fibroma	2 (4%)
Complete resection, *n* (%)	66 (100%)
*En bloc* resection, *n* (%)	64 (97%)
Procedure time (minutes), median (range)	46 (13–224)
<60 min, *n* (%)	39 (59%)
≥60 min, *n* (%)	27 (41%)
**Complications, *n* (%)**	
No	56 (85%)
Yes	10 (15%)
**Complication type, *n* (%)**	
Subcutaneous emphysema	2 (3%)
Pneumomediastinum	1 (2%)
Pneumothorax	2 (3%)
Mucosal injury	4 (6%)
Esophageal-pleural fistula	1 (2%)
Hospital stays (days), median (range)	5 (3–9)
<6 days, *n* (%)	49 (74%)
≥6 days, *n* (%)	17 (26%)
Follow-up period (month), median (range)	36 (28–51)
Residual, *n* (%)	0 (0%)
Recurrence, *n* (%)	0 (0%)

Data are median (range), or *n* (%). SMT, submucosal tumors; STER, submucosal tunnel endoscopic resection.

### Resection rate and procedure time

Complete resection and *en bloc* resection of the tumor were achieved in 66 patients (100%) and 64 patients (97%), respectively. Notably, the tumor size was more than 25 mm in two patients *en bloc* resection was not achieved. The median overall time for the STER procedure was 45 min (range: 13–224 min). Twenty-seven patients (41%) had a procedure time ≥60 min. In the univariable logistic regression analysis, tumor size, tumor layer, tumor shape, tumor histopathology, tunnel length, and occurrence of complications were associated with longer operative times (≥60 min). In the multivariable analysis, only tumor size (OR 1.37, 95% CI 1.13–1.67; *p* = 0.001) and tumor histopathology (other SMTs vs. leiomyomas: OR 24.34, 95% CI 3.19–185.80; *p* = 0.002) were identified as independent predictors of longer operative times (Table 2).

**TABLE 2:** Univariable and multivariable logistic regression analysis of factors associated with long operative times (≥60 min) in 66 patients with upper gastrointestinal submucosal tumors (SMTs) treated with submucosal tunnel endoscopic resection (STER).

Variables	Operative times		Univariable analysis		Multivariable analysis	
	**<60 min (*N* = 39)**	**≥60 min (*N* = 27)**	**OR (95% CI)**	***P***	**OR (95% CI)**	***P***
Age (years)	53 (29–73)	53 (27–64)	0.96 (0.91–1.01)	0.132		
**Gender**						
Male	19 (49%)	13 (48%)	Ref.			
Female	20 (51%)	14 (52%)	1.02 (0.38–2.78)	0.965		
Tumor size (mm)	15 (6–30)	30 (12–75)	1.24 (1.10–1.40)	**< 0.001**	1.37 (1.13–1.67)	0.001
**Tumor location**						
Esophagus	24 (62%)	10 (37%)	Ref.			
Esophagogastric junction	11 (28%)	13 (48%)	2.77 (0.93–8.61)	0.067		
Stomach	4 (10%)	4 (15%)	2.34 (0.45–12.4)	0.306		
**Tumor layer**						
Muscularis mucosa or submucosa	11 (28%)	2 (7%)	Ref.			
Muscularis propria	28 (72%)	25 (93%)	4.56 (1.07–34.6)	**0.040**		
**Tumor shape**						
Regular	35 (90%)	16 (59%)	Ref.			
Irregular	4 (10%)	11 (41%)	5.72 (1.65–24.1)	**0.005**		
Tunnel length (cm)	3 (2–6)	3 (1–5)	2.02 (0.94–4.35)	**0.071**		
**Histopathology**						
Leiomyomas	33 (85%)	17 (63%)	Ref.			
Other SMTs	6 (15%)	10 (37%)	3.15 (0.98–10.9)	**0.054**	24.34 (3.19–185.80)	0.002
**Complications**						
No	36 (92%)	20 (74%)	Ref.			
Yes	3 (8%)	7 (26%)	4.01 (0.97–21.4)	**0.056**		

SMT, submucosal tumors; STER, submucosal tunnel endoscopic resection; OR, odds ratio; CI, confidence intervals.

Variables in bold were included in subsequent multivariable analysis.

### STER-related complications

STER-related complications were observed in 10 patients (15%), including subcutaneous emphysema in two (3%), pneumomediastinum in one (2%), pneumothorax in two (3%), mucosal injury in four (6%), and esophageal-pleural fistula in one (2%). No patients developed delayed bleeding, GI tract leakage, or secondary peritoneal/abdominal infections. All patients with STER-related complications were treated successfully using conservative treatment, and there were no treatment-related deaths. In the univariable logistic regression analysis, tumor size and procedure time were associated with the occurrence of complications. In the multivariable analysis, only tumor size (OR 1.06, 95% CI 1.01–1.10; *p* = 0.012) was identified as the risk factor for complications (Table 3).

**TABLE 3:** Univariable and multivariable logistic regression analysis of factors associated with complications in 66 patients with upper gastrointestinal submucosal tumors (SMTs) treated with submucosal tunnel endoscopic resection (STER).

Variables	Complications		Univariable analysis		Multivariable analysis	
	**No (*n* = 56)**	**Yes (*n* = 10)**	**OR (95% CI)**	***P***	**OR (95% CI)**	***P***
Age (years)	53 (29–73)	46 (27–64)	0.95 (0.88–1.01)	0.116		
**Gender**						
Male	29 (52%)	3 (30%)	Ref.			
Female	27 (48%)	7 (70%)	2.41 (0.59–12.8)	0.229		
Tumor size (mm)	18 (6–75)	35 (12–60)	1.06 (1.01–1.10)	**0.012**	1.06 (1.01–1.10)	0.012
**Tumor location**						
Esophagus	29 (52%)	5 (50%)	Ref.			
Cardia	20 (36%)	4 (40%)	1.16 (0.25–5.12)	0.841		
Stomach	7 (12%)	1 (10%)	0.91 (0.03–7.35)	0.937		
**Tumor layer**						
Muscularis mucosa or submucosa	12 (21%)	1 (10%)	Ref.			
Muscularis propria	44 (79%)	9 (90%)	2.19 (0.35–58.5)	0.458		
**Tumor shape**						
Regular	45 (80%)	6 (60%)	Ref.			
Irregular	11 (20%)	4 (40%)	2.70 (0.58–11.6)	0.197		
Tunnel length (cm)	3 (1–6)	3 (2–5)	1.83 (0.84–4.02)	0.130		
**Histopathology**						
Leiomyomas	42 (75%)	8 (80%)	Ref.			
Other SMTs	14 (25%)	2 (20%)	0.79 (0.10–3.74)	0.783		
Procedure time (minutes)	38 (13–177)	79 (25–224)	1.02 (1.01–1.03)	**0.004**		

SMT, submucosal tumors; STER, submucosal tunnel endoscopic resection; OR, odds ratio, CI, confidence intervals.

Variables in bold were included in subsequent multivariable analysis.

### Hospital time

The median hospital stay was 5 days (range: 3–9 days). Seventeen patients (26%) had a hospital stay of ≥6 days, while the hospital stay was less than 6 days in the remaining 49 patients (74%). In the univariable logistic regression analysis, gender, tumor size, procedure time, and occurrence of complications were associated with longer hospital times. In the multivariable analysis, only tumor size (OR 1.05, 95% CI 1.01–1.09; *p* = 0.035) and occurrence of complications (OR 6.94, 95% CI 1.40–34.54; *p* = 0.018) were independently associated with longer hospital times (Table 4).

**TABLE 4:** Univariable and multivariable logistic regression analysis of factors associated with long hospital times (≥6 days) in 66 patients with upper gastrointestinal submucosal tumors (SMTs) treated with submucosal tunnel endoscopic resection (STER).

Variables	Hospital time		Univariable analysis		Multivariable analysis	
	**<6 d (*n* = 49)**	**≥6 d (*n* = 17)**	**OR (95% CI)**	***P***	**OR (95% CI)**	***P***
Age (years)	51 (29–73)	54 (27–64)	1.02 (0.96–1.09)	0.453		
**Gender**						
Male	27 (55%)	5 (29%)	Ref.			
Female	22 (45%)	12 (71%)	2.86 (0.90–10.4)	**0.077**		
Tumor size (mm)	15 (6–60)	30 (10–75)	1.06 (1.02–1.11)	**0.005**	1.05 (1.01–1.09)	0.035
**Tumor location**						
Esophagus	25 (51%)	9 (53%)	Ref.			
Esophagogastric junction	19 (39%)	5 (29%)	0.74 (0.19–2.57)	0.643		
Stomach	5 (10%)	3 (18%)	1.67 (0.27–8.69)	0.557		
**Tumor layer**						
Muscularis mucosa or submucosa	10 (20%)	3 (18%)	Ref.			
Muscularis propria	39 (80%)	14 (82%)	1.16 (0.29–6.07)	0.840		
**Tumor shape**						
Regular	39 (80%)	12 (71%)	Ref.			
Irregular	10 (20%)	5 (29%)	1.63 (0.42–5.71)	0.463		
Tunnel length (cm)	3 (1–6)	3 (2–5)	1.75 (0.85–3.61)	0.128		
**Histopathology**						
Leiomyomas	37 (76%)	13 (76%)	Ref.			
Other SMTs	12 (24%)	4 (24%)	0.97 (0.23–3.43)	0.959		
Procedure time (minutes)	35 (13–195)	63 (25–224)	1.02 (1.00–1.03)	**0.014**		
**Complications**						
No	46 (94%)	10 (59%)	Ref.		Ref.	
Yes	3 (6%)	7 (41%)	9.96 (2.28–56.1)	**0.002**	6.94 (1.40–34.54)	0.018

SMT, submucosal tumors; STER, submucosal tunnel endoscopic resection; OR, odds ratio; CI, confidence intervals.

Variables in bold were included in subsequent multivariable analysis.

### Follow-up results

The overall median follow-up period was 36 months (range: 28–51 months). No residual tumor or tumor recurrence was observed during the follow-up period.

### Risk stratification based on tumor size

**FIGURE 3:**
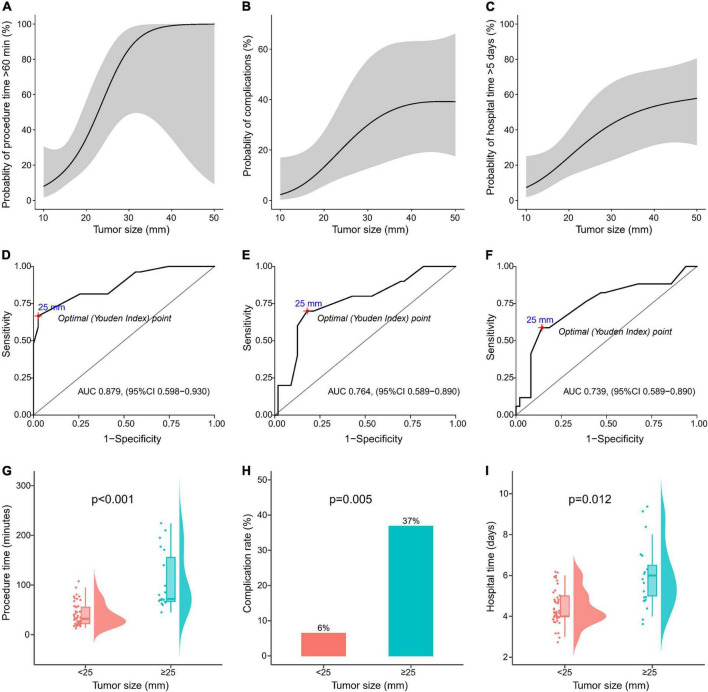
Risk stratification based on tumor size. Restricted cubic splines showing the association of tumor size with the probability of **(A)** long procedure time (≥60 min), **(B)** occurrence of procedure-related complications, and **(C)** long hospital stay (≥6 days) in patients with gastrointestinal submucosal tumors undergoing submucosal tunnel endoscopic resection (STER). Receiver operating characteristic (ROC) curves showing discrimination of tumor size for **(D)** long procedure time (≥60 min), **(E)** occurrence of procedure-related complications, and **(F)** long hospital stay (≥6 days). The optimal cutoff value of tumor size with the largest value of the Youden index (sensitivity + specificity–1) was identified based on the ROC curve. Comparisons between patients with a tumor size of ≥25 mm and those without a tumor size of <25 mm in terms of **(G)** procedure time, **(H)** rate procedure-related complications, and **(I)** hospital stay time.

The association of tumor size and the risk of long procedure time (≥60 min), STER-related complications, and long hospital stays are shown in Figures 3A–C. The risk of long procedure time (≥60 min), STER-related complications, and long hospital stay were increased with the increasing tumor size. As shown in Figures 3D–F, the discrimination (the ability of an index to differentiate between patients who do and do not experience an event) of tumor size was good for the longer procedure time (AUC 0.879, 95% CI 0.598-0.930), occurrence of complication (AUC 0.764, 95% CI 0.589-0.890), and long hospital stay (AUC 0.739, 95% CI 0.589-0.890).

The ROC curves identified a tumor size of 25 mm as the cutoff with the greatest sensitivity and specificity. With this threshold, 19 patients (29%) had a tumor size of ≥25 mm and 47 patients (71%) had a tumor size of <25 mm. The clinicopathological characteristics of patients grouped according to tumor size of 25 mm are shown in Table 5. The procedure time (106.8 ± 58.6 vs. 40.2 ± 23.2 min, *p* < 0.001) (Figure 3G) as well as hospital time (5.9 ± 1.5 vs. 4.4 ± 0.9 days, *p* = 0.012) was longer, and the incidence of complications (37 vs. 6%, *p* = 0.005) was higher in patients with a tumor size ≥25 mm compared with those with a tumor size <25 mm (Figures 3H, I). Furthermore, patients with a tumor size ≥25 mm had 76-fold (95 vs. 19%; OR 76.00, 95% CI 8.94–646.44; *p* < 0.001) risk of long procedure time, 8.56-fold (37 vs. 6%; OR 8.56, 95% CI 1.92–38.17; *p* = 0.005) risk of occurrence of complications, and 6.35-fold (53 vs. 15%; OR 6.35, 95% CI 1.90–21.22; *p* = 0.003) risk of long hospital stay.

**TABLE 5:** Comparison of the clinicopathological characteristics according to tumor size <25 mm or ≥25 mm in patients with upper gastrointestinal submucosal tumors (SMTs) treated with submucosal tunnel endoscopic resection (STER).

Characteristics	Tumor size < 25 mm (*n* = 47)	Tumor size ≥ 25 mm (*n* = 19)	*P*
Gender, *n* (%)			0.698
Male	24 (51%)	8 (42%)	
Female	23 (49%)	11 (58%)	
Age (years), median (range)	53 (29–73)	53 (27–64)	0.212
Tumor size (mm), median (range)	15 (6–20)	40 (25–75)	<0.001
Tumor Location, *n* (%)			0.515
Upper esophagus	2 (4%)	2 (11%)	
Middle esophagus	6 (13%)	2 (11%)	
Lower esophagus	18 (38%)	4 (21%)	
Esophagogastric junction	15 (32%)	9 (47%)	
Stomach	6 (13%)	2 (11%)	
Tumor layer, *n* (%)			0.003
Muscularis mucosa	11 (23%)	0 (0%)	
Submucosa	0 (0%)	2 (11%)	
Muscularis propria	36 (77%)	17 (89%)	
Tumor shape, *n* (%)			0.001
Regular	42 (89%)	9 (47%)	
Irregular	5 (11%)	10 (53%)	
Tunnel length (cm), median (range)	3 (2–6)	3 (1–5)	0.009
Complete resection, *n* (%)	47 (100%)	19 (100%)	
*En bloc* resection, *n* (%)	47 (100%)	17 (89%)	0.080
Histopathology, *n* (%)			0.093
Leiomyomas	35 (74%)	15 (79%)	
Gastrointestinal stromal tumors	10 (21%)	1 (5%)	
Lipomas	0 (0%)	1 (5%)	
Schwannoma	1 (2%)	1 (5%)	
Fibroma	1 (2%)	0 (0%)	
Granuloma	0 (0%)	1 (5%)	
Procedure time (minutes), median (range)	32 (13–107)	72 (45–224)	<0.001
<60 min, *n* (%)	38 (81%)	1 (5%)	
≥60 min, *n* (%)	9 (19%)	18 (95%)	
Complications, *n* (%)	3 (6%)	7 (37%)	0.004
Subcutaneous emphysema	1 (2%)	1 (5%)	
Pneumomediastinum	1 (2%)	0 (0%)	
Pneumothorax	0 (0%)	2 (11%)	
Mucosal injury	0 (0%)	4 (21%)	
Esophageal-pleural fistula	1 (2%)	0 (0%)	
Hospital time, median (range)	4 (3–6)	6 (4–9)	<0.001
<6 days, *n* (%)	40 (85%)	9 (47%)	
≥6 days, *n* (%)	7 (15%)	10 (53%)	

SMT, submucosal tumors; STER, submucosal tunnel endoscopic resection.

## Discussion

In this observational study, we found that (i) STER is effective and safe for the treatment of upper gastrointestinal SMTs, with a complete resection rate of 100%, an *en bloc* resection rate of 97%, and a STER-related complication rate of 15%; (ii) greater tumor size was associated with longer procedure time, higher risk of STER-related complications, and longer hospital stay; (iii) with a cutoff value of tumor size ≥25 mm, patients with upper gastrointestinal SMTs treated with STER can be classified as low- and high-risk groups.

The complete resection rate (100%) and *en bloc* resection rate (97%) in our study were comparable with those reported in previous studies (7, 10–12), confirming that STER is an effective and safe technique for the treatment of upper gastrointestinal SMTs. In a large cohort by Chen et al. (6), the *en bloc* resection was achieved in 90.6% of patients; tumors with irregular shape and greater size were significantly associated with piecemeal resection. In our results, *en bloc* resection was achieved in only two patients. Thus, univariable and multivariable analyses were not performed. However, in two patients *en bloc* resection was not achieved, the tumor size was more than 25 mm, which confirms that tumor size is significantly associated with the efficacy of *en bloc* resection.

The reported STER-related complications in patients with upper gastrointestinal SMTs varied from 0 to 40% in different studies. The incidence of STER-related complications (15%) in our patients was within the reported range (5, 7, 8, 13–16). Consistent with previous studies, the common complications associated with STER were air leakage symptoms and perforation (5, 8, 17, 18). All STER-related complications were cured without intervention or treated conservatively without the need for surgery, which further proved the safety of STER.

In addition, we found that larger tumor size was associated with longer procedure time, higher risk of STER-related complications, and longer hospital stay. In previous studies (5, 7, 8, 13–16), although other factors were also reported with those outcomes, tumor size is the most constantly reported factor. Indeed, in clinical practice, larger tumors are more technically demanding and need more time to resect. Because of the limited space in the established submucosal tunnel, it has been suggested by the Chinese society of digestive endoscopy that the implementation of STER for SMTs with a transverse diameter of ≤3.5 cm can facilitate a high *en bloc* resection rate (19).

In terms of risk stratification, our data showed that using tumor size with a threshold of 25 mm is useful for identifying patients with a low and high risk of longer procedure time, higher risk of STER-related complications, and longer hospital stays. Patients with a tumor size of ≥25 mm had 76-fold (95% CI 8.94–646.44; *p* < 0.001) risk of long procedure time, 8.56-fold (95% CI 1.92–38.17; *p* = 0.005) risk of occurrence of complications, and 6.35-fold (95% CI 1.90–21.22; *p* = 0.003) risk of long hospital stay. The AUC of 0.879 for longer procedure time, 0.764 for the occurrence of STER-related complications, and 0.739 for a long hospital stay further suggests the good discrimination of tumor size. Thus, considering the high risk of longer procedure time, higher risk of STER-related complications, and longer hospital stay for patients with large tumors, the STER procedure should be performed by a more experienced hand or other treatment methods should be adopted. Furthermore, the tumor size has the advantages of objectiveness, easy-to-get, and easy-to-use; thus, it may provide risk stratification criteria to control heterogeneity in future clinical trials.

Although the predictive value of tumor size seems obvious, the cutoff values for risk stratification have long been controversial, and most researchers featuring large tumors adopted arbitrary or empirical cutoffs. The recommended maximum resectable lesion size by most researchers is less than 35 mm in diameter because large tumors could cause loss of endoscopic visualization in a limited submucosal space (5, 19–21). However, the successful STER treatment of larger tumors was not uncommonly reported (3, 18). Furthermore, it should be noted that purpose of our study was to identify risk factors of longer procedure time, higher risk of STER-related complications, and longer hospital stay instead of maximum resectable lesion size. Therefore, the cutoff value of tumor size was smaller than 35 mm.

The present study has several limitations which should be considered. First, it was designed as a retrospective study with relatively small sample size. Second, control groups were deficient for the comparison of outcomes with those of other therapies such as ESE and EFR. Third, data on the patients’ comorbidities and their pharmacological history were not available in our study, which may influence the accuracy of risk stratification. Finally, the follow-up time in the present study was short. Future studies are needed to validate the results observed.

In conclusion, STER is an effective and safe technique for the treatment of upper gastrointestinal SMTs. The tumor size of ≥25 mm was associated with longer procedure time, higher risk of STER-related complications, and longer hospital stay. Further prospective studies in comparison with other endoscopic procedures and surgical treatments are needed.

## Data availability statement

The raw data supporting the conclusions of this article will be made available by the authors, without undue reservation.

## Ethics statement

The studies involving human participants were reviewed and approved by the Ethics Committee of Xijing Hospital. The patients/participants provided their written informed consent to participate in this study. Written informed consent was obtained from the individual(s) for the publication of any identifiable images or data included in this article.

## Author contributions

YL and HX: study concept and design, analysis, and interpretation of data. YL: drafting of the manuscript and statistical analysis. XL and HX: critical revision of the manuscript for important intellectual content. All authors performed acquisition of data, contributed to the article, and approved the submitted version.

## Footnotes

1
http://www.R-project.org/
